# Dynamic and static ultrasound features predictive of vesicoureteral reflux and renal damage in children and adolescents with neurogenic bladder

**DOI:** 10.1590/S1677-5538.IBJU.2023.0311

**Published:** 2024-02-07

**Authors:** Carlos Magno Paiva da Silva, Mônica Maria de Almeida Vasconcelos, Eleonora Moreira Lima, José de Bessa, Otávio Augusto Fonseca Reis, Maria Francisca Tereza Freire Filgueiras, Roberta Vasconcellos Menezes de Azevedo, José Murillo Bastos, Eduardo Araújo Oliveira, Flávia Cristina de Carvalho Mrad

**Affiliations:** 1 Faculdade de Medicina Universidade Federal de Minas Gerais Departamento de Pediatria e Unidade de Nefrologia Pediátrica Belo Horizonte MG Brasil Departamento de Pediatria e Unidade de Nefrologia Pediátrica, Faculdade de Medicina Universidade Federal de Minas Gerais – UFMG, Belo Horizonte, MG, Brasil; 2 Universidade Estadual de Feira de Santana Departamento de Urologia Feira de Santana BA Brasil Departamento de Urologia, Universidade Estadual de Feira de Santana – UEFS, Feira de Santana, BA, Brasil; 3 Universidade Federal de Juiz de Fora Departamento de Urologia Faculdade de Medicina Juiz de Fora MG Brasil Departamento de Urologia, Faculdade de Medicina, Universidade Federal de Juiz de Fora – UFJF, Juiz de Fora, MG, Brasil; 4 Faculdade de Ciências Médicas de Juiz de Fora e Maternidade Therezinha de Jesus Departamento de Urologia Juiz de Fora MG Brasil Departamento de Urologia, Faculdade de Ciências Médicas de Juiz de Fora e Maternidade Therezinha de Jesus, Juiz de Fora, MG, Brasil

**Keywords:** Urinary Bladder, Neurogenic, Meningomyelocele, Vesico-Ureteral Reflux

## Abstract

**Purpose:**

This study aimed to analyze the diagnostic accuracy of dynamic and static ultrasound (DSUS) in detecting vesicoureteral reflux (VUR) and renal scarring in a cohort of children with neurogenic bladder (NB).

**Materials and Methods:**

A retrospective, longitudinal, observational study was conducted using the Reporting Diagnostic Accuracy Studies guideline. The DSUS (index test) data were compared with voiding cystourethrography (VCUG) and renal scintigraphy 99mTc-dimercaptosuccinic (reference tests). Overall performance for predicting VUR and renal scarring was assessed using renal pelvic diameter (RPD)/distal ureteral diameter and renal parenchymal thinning on DSUS, respectively.

**Results:**

A total of 107 patients (66 girls, median age 9.6 years) participated. Seventeen patients (15.9%) presented VUR, eight bilateral. For overall reflux grade, the AUC was 0.624 for RPD and 0.630 for distal ureteral diameter. The diagnostic performance for detecting high-grade VUR was slightly better for DSUS parameters. The AUC was 0.666 for RPD and 0.691 for distal ureteral diameter. The cut-offs of 5 mm for RPD and 6.5 mm for distal ureteral diameter presented the best diagnostic odds ratio (DOR) to identify high-grade VUR. The increase of RPD during detrusor contractions showed an accuracy of 89.2%. The thinness of renal parenchyma presented an accuracy of 88% for renal scarring.

**Conclusion:**

DSUS predicts VUR and renal scarring in children with NB with fair to good accuracy, and all measurements exhibited a high negative predictive value (NPV). The increase in RPD during voiding or detrusor contractions proved to be the most accurate parameter for indicating the presence of VUR in this study.

## INTRODUCTION

The most common cause of neurogenic bladder (NB) in children is neurospinal dysraphism (1-3). NB is present in up to 98% of children with myelomeningocele (4). About 25% of the most severe symptoms in pediatric urology are associated with neurogenic bladder (5), and 40% of children with NB develop chronic kidney disease (6). Patients with NB may present with various patterns of detrusor-sphincter dyssynergia and increased intravesical pressure, which can lead to urinary and/or fecal incontinence, urinary tract infections (UTIs), vesicoureteral reflux (VUR) and renal impairment (1, 3, 7). The diagnosis and follow-up of patients with NB involves a multidisciplinary approach, including serial clinical, laboratory, and imaging tests. The goals of managing bladder dysfunction in children are maintaining a low-pressure, high-compliance bladder, and preventing upper urinary tract deterioration (8).

VUR, an important risk factor for pyelonephritis and renal scarring (1, 7, 9, 10), is present in up to one-third of children with NB (8), making its diagnosis and approach essential (3, 8). VCUG and renal scintigraphy are the gold standard tests for diagnosing VUR and renal scarring (9, 11, 12). The role of renal and bladder ultrasound as a screening tool for VUR and kidney damage in children with NB has been debated (13). However, in this sense, the lack of US accuracy for VUR or renal scarring may hinder its use in NB, given the need to prevent irreversible renal damage (3, 9). However, the development of the dynamic and static ultrasound (DSUS) technique made it possible to obtain essential data for the diagnosis and follow-up of patients with NB (14). We hypothesize that the magnitude of specific DSUS measurements could predict the presence of VUR and renal scars. Therefore, this study aims to analyze the diagnostic accuracy of DSUS in detecting VUR and kidney damage in our cohort of children and adolescents with NB.

## MATERIALS AND METHODS

### Ethical approval

The study was approved by Institutional Review Committee (IRB) under protocol CAAE 37450820000005149, position statement number 4.487.114. Legal guardians and participants aged 10 and 17 signed the Informed Consent Term and the Assent Term, respectively. The medical records were selected through an active search in the Medical Service and Archive after the institution's consent and signature of the Data Use Commitment Term.

### Study design and patients

This retrospective cohort study included 127 consecutive patients enrolled in the Multidisciplinary Outpatient Clinic for Children and Adolescents with NB. We designed our study and reported our findings following the STAndards for the Reporting of Diagnostic accuracy studies (STARD) presented in Supplement 1 (15). Eligibility criteria were all patients with NB enrolled in the service between 1997 to 2022 who underwent DSUS, VCUG and renal scintigraphy (99mTc-DMSA) according to the care protocol. Twenty patients were excluded from the analysis: 15 due to lack of information in the medical records, and five refused to participate in the study.

### Study protocol

A systematic clinical protocol was applied to all NB patients enrolled in the multidisciplinary outpatient clinic (1-3). On admission, we performed a clinical laboratory and imaging investigation (DSUS, urodynamic study, VCUG to assess VUR, and renal scintigraphy (99mTc-DMSA) to diagnose NB status and assess renal scarring. Our follow-up protocol included clinical examination, laboratory analysis at semi-annual intervals, and DSUS, annually or as clinically necessary.

### Index test

DSUS was considered the index test (test being evaluated) and was performed by the same trained examiner using a standard method (14). The exams were performed, on annual basis, using a Toshiba/Canon^®^ Prima SLC Ultrasound Device, model Aplio 300 or 400 supplied with multifrequency convex (3.7 to 7.6 MHz), linear (8.0 to 12.0 MHz), high frequency electronic linear (13.0 to 18.0 MHz) transducers.

**Figure 1:**
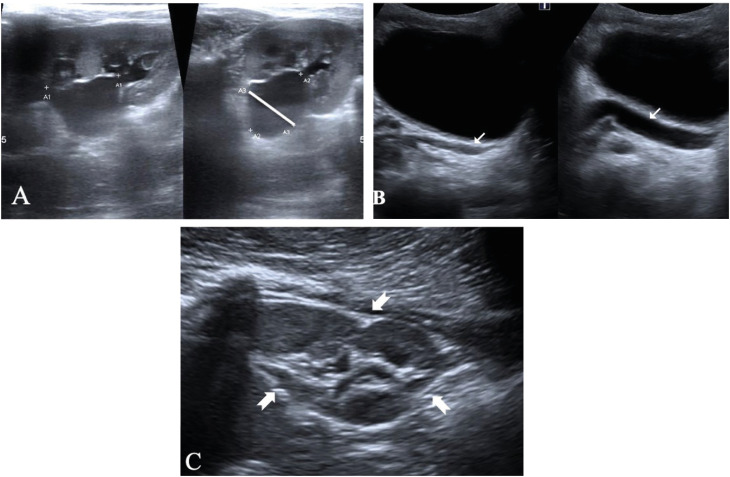
Sonography indexes. (A- renal pelvic diameter, B- distal ureteral diameter, C-renal scarring).

The assessed index tests were based on the first ultrasound after enrollment in the outpatient clinic. For patients with bilateral urinary tract alteration, index tests were generated for each renal unit. The following sonography indexes were determined as proposed by Dinkel et al. (16): Renal pelvic diameter (RPD) was determined by the greatest anteroposterior diameter of the renal pelvis acquired in a transverse plane on ultrasound dorsal images; Distal ureter diameter; Renal parenchyma thickness (RPT) was measured at the transverse view for each kidney; Bladder wall thickening; Bladder capacity; Presence of bladder residual urine (absent, mild, severe); Bladder trabeculation; Renal scarring. Renal scarring on DSUS was assessed using the following criteria: proximity of sinus echoes to the cortical surface, loss of pyramids, irregular outline, and loss of definition of capsular echo (17). In addition, in DSUS, we evaluated RPD increase during voiding or detrusor contractions as an indirect indicator of the presence of VUR (14) (Figure-1).

### Reference tests

Examiners who were unaware of the other tests' results performed the index and standard reference tests.

The VCUG was considered the reference standard for VUR. The VCUG was requested at the beginning of the follow-up with a maximum interval of six months concerning the DSUS. We used the classification of VUR proposed by the Reflux Study Committee (18). In addition, according to reflux grade, we classified reflux as low-grade (I), mild to moderate-grade (II to III), and high-grade (IV-V) (19).

The reference test for renal scarring was renal scintigraphy (99mTc-DMSA), performed on admission (after the fourth month of life for infants) and later according to a clinical decision (episodes of recurrent UTIs/pyelonephritis) (1-3, 7).

### Statistical Analysis

Continuous data were recorded as median and 25th to 75th interquartile range (IQ). The nonparametric Mann-Whitney test was used to compare these variables. Dichotomous variables were compared using the 2-sided chi-square test.

The diagnostic accuracy of the indexes tests was assessed by sensitivity, specificity, positive predictive value (PPV), negative predictive value (NPV), and likelihood ratios (LR). Receiver-operating characteristic (ROC) curves were analyzed for the overall diagnostic accuracy of continuous indexes (RPD and distal ureteral diameter) in discriminating infants who will present the events of interest (VUR and renal scarring). The area under the curve (AUC) was interpreted as the probability that a randomly selected patient with the event of interest had a larger maximum diameter than a randomly selected patient without the event of the interest.

We also analyzed the combined results of the continuous indexes (RPD and distal ureteral diameter) (20). Therefore, two possibilities were analyzed, using the "OR" rule, i.e., considering a positive diagnosis if either test was positive and a negative diagnosis if both tests were negative, and the "AND" rule, i.e., considering a positive diagnosis only if both tests were positive and a negative diagnosis if either test was negative.

## RESULTS

A total of 107 patients (66 females) were included in the analysis. The main baseline clinical characteristics of patients are summarized in Table-1. Seventeen patients (15.9%) presented VUR, eight bilateral, giving a total of 25 refluxing units (11 mild to moderate grade (II-III) and 14 high-grade (IV-V) reflux).

**Table 1:** Patient clinical and demographics characteristics.

		N = 107
**Gender**		
	Male	41 (38.3)
	Female	66 (61.7)
**Neural tube defect**		
	Spina bifida	92 (86.0)
	Others	15 (14.0
**Age (years)**		
	Median	9.6
	Interquartile range	6.1 – 17.0
	Mean	11.6
	Standard deviation	6.5
**Vesicoureteral reflux**		
	Absent	90 (84.1)
	Unilateral	9 (8.4)
	Bilateral	8 (7.5)
**Vesicoureteral reflux (units. grade)**		
	Absent	189 (88.3)
	Mild Moderate (II III)	11 (5.1)
	Severe (IV V)	14 (6.5)
**Renal damage (99mTc-DMSA)**		
	Absent	72 (80.2)
**Unilateral**		18 (19.6)
	Bilateral	2 (2.2)

99mTc-DMSA renal scintigraphy 99mTc-dimercaptosuccinic

### Vesicoureteral reflux

**Figure 2:**
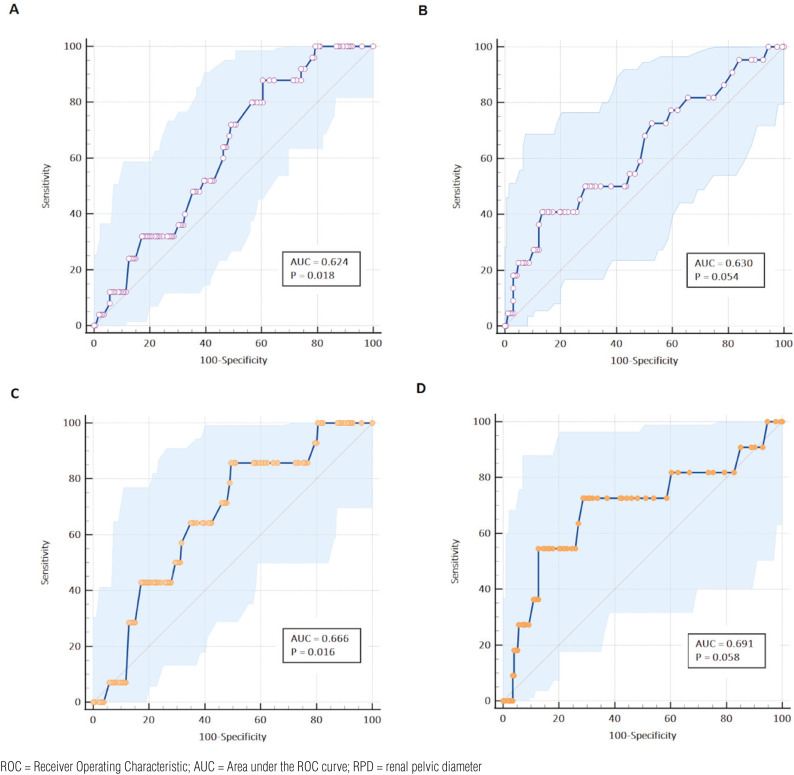
ROC curves comparing ultrasonographic measurements of RPD and the presence of general vesicoureteral reflux. (A- RPD, B- distal ureteral diameter) and high-grade vesicoureteral reflux (C- RPD, B- distal ureteral diameter).

The diagnostic accuracy of DSUS in predicting reflux was evaluated using the maximum RPD and the maximum distal ureteral diameter. For overall reflux grade, the AUC was 0.624 (95% CI, = 0.553 - 0.692) for RPD and 0.630 (95% CI, 0.556 – 0.700) for distal ureteral diameter (Figures 1A-B). The diagnostic performance for detecting high-grade VUR (Grade IV-V) was slightly better for both US parameters. The AUC was 0.666 (95% CI, 0.596 - 0.731) for RPD and 0.691 (95% CI, 0.619 – 0.757) for distal ureteral diameter (Figure-2).

Table-2 shows the diagnostic performance of various RPD and distal ureteral diameter thresholds indicating VUR (Grade II-V). The cut-offs of 5 mm for RPD and 6.5 mm for distal ureteral diameter presented the best diagnostic odds ratio (DOR) to identify children with high-grade VUR. The combined results of both pelvic and ureter diameters are also shown in Table-2. Regarding both tests in parallel and using the "OR rule," i.e., the sensitivity increased to 80% (95% CI, 59.3-93.2), and the negative predictive value (NPV) to 94.8% (95% CI, 89.8-97.6). However, considering the low prevalence of reflux, the positive predictive value (PPV) was only 17.4% (95% CI, 7.8-16.9). By contrast, using the "AND rule", the specificity increased to 88.7% (95% CI, 83.3-92.9). However, again the PPV was only 27.6% (95% CI, 15.9-43.4), but the NPV was 90.7% (95% CI, 88.1-92.8).

**Table 2:** Diagnostic accuracy of sonography measurements for vesicoureteral reflux (grade II- V)

		Sensitivity% (95%CI)	Specificity% (95%CI)	PPV	NPV	DOR
**RPD cut-offs**						
	> 5.0 mm	72.0 (50.6 - 87.9)	49.4 (41.5 - 56.8)	16.8 (13.2 - 21.2)	92.5 (86.6 - 95.9)	2.5
	> 7.5 mm	36.0 (18.0 - 57.5)	68.0 (60.5 - 74.8)	13.8 (8.4 - 22.1)	88.1 (84.5 - 91.0)	1.2
	> 10.0 mm	32.0 (14.9 - 53.5)	79.4 (72.7 - 85.2)	18.2 (10.5 - 29.7)	89.1 (86.1 - 91.5)	1.8
	> 12.5 mm	24.0 (9.4 - 45.1)	86.3 (80.3 - 91.0)	20.0 (10.3 - 35.3)	88.8 (86.4 - 90.9)	2.0
**Ureter cut-offs**						
	> 4.5 mm	50.0 (28.2 - 71.8)	68.7 (61.0 - 75.7)	17.7 (11.8 - 25.8)	91.1 (86.9 - 94.0)	2.2
	> 5.5 mm	40.9 (20.7 - 63.6)	77.3 (70.1 - 83.5)	19.6 (12.0 - 30.2)	90.6 (87.1 - 93.3)	2.4
	> 6.5 mm	40.9 (20.7 - 63.6)	86.5 (80.3 - 91.3)	29.0 (17.8 - 43.6)	91.6 (88.4 - 93.9)	4.5
	> 7.5 mm	27.3 (10.7 - 50.2)	89.0 (83.1 - 93.3)	25.0 (12.9 - 42.8)	90.1 (87.5 - 92.2)	3.0
**Combined indexes (OR rule)**						
	RPD > 5.0 mm	80.0	49.2	17.4	94.8	3.9
	Ureter > 6.5 mm	(59.3-93.2)	(41.8 - 56.6)	(7.8 – 16.9)	(89.8 – 97.6)	
**Combined indexes (AND rule)**						
	RPD > 5.0 mm	32.0	88.7	27.6	90.7	3.7
	Ureter > 6.5 mm	(14.9-53.5)	(83.3 – 92.9)	(15.9 - 43.4)	(88.1 - 92.8)	

95% CI = Confidence Interval 95%; PPV = positive predictive value; NPV = negative predictive value; DOR = diagnostic odds ratio

Table-3 shows a similar analysis for high-grade VUR (Grade IV-V). Of note, the same cutoff points of 5 mm for RPD and 6.5 mm for distal ureteral diameter had the best DOR to identify children with high-grade reflux. Regarding the two tests in parallel, using the "OR rule", the sensitivity increased to 92.9% (CI 95%, 66.1-99.8) and the NPV to 94.8% (CI 95%, 93.5-99.8), the specificity increased to 89.5% (95% CI, 81.8-91.9). It still shows the diagnostic performance of categorical variables indicating high-grade VUR (Grade IV-V). Overall performance was relatively poor for all measurements, except for the observation, in the dynamic phase of the test, of an increase in RPD during urination, a finding that had a specificity of 92.5% (95% CI, 87.9-95.7), NPV of 95.9% (95% CI, 93.6-97.3) and an accuracy of 89.2% (95% CI, 84.3-93.0) for reflux high-grade.

**Table 3:** Diagnostic accuracy of sonography measurements for high-grade vesicoureteral reflux (grade IV- V).

		Sensitivity% (95%CI)	Specificity% (95%CI)	PPV (95%CI)	NPV (95%CI)	DOR
**RPD cut-offs**					
	> 5.0 mm	85.7 (57.2 - 98.2)	48.9	11.2	97.8	5.8
	> 7.5 mm	50.0 (23.0 - 77.0)				2.2
	> 10.0 mm	42.9 (17.7 - 71.1)				2.9
	> 12.5 mm	28.6 (8.4 - 58.1)				2.5
**Ureter cut-offs**						
	> 4.5 mm	72.7(39.0 - 94.0)	68.9 (61.5 - 75.7)	12.9 (8.8 - 18.5)	97.6 (93.8 - 99.1)	5.8
	> 5.5 mm	54.5 (23.4 - 83.3)	77.0(70.0 - 83.0)	13.0 (7.6 - 21.5)	96.4 (93.3 - 98.1)	4.0
	> 6.5 mm	54.5 (23.4 - 83.3)	85.6 (79.5 - 90.5)	19.4 (11.1 - 31.5)	96.8 (94.0 - 98.3)	7.2
	> 7.5 mm	36.4 (10.9 - 69.2)	88.5 (82.8 - 92.8)	16.7 (7.6 - 32.6)	95.7 (93.3 - 97.2)	4.4
**Combined indexes (OR rule)**						
	RPD > 5.0 mm	92.9	48.5	11.3	98.9	12.3
	Ureter > 6.5 mm	(66.1 - 99.8)	(41.3 -55.7)	(9.5 - 13.4)	(93.5 - 99.8)	
**Combined indexes (AND rule)**						
	RPD > 5.0 mm	42.8	89.5	20.7	95.3	5.2
	Ureter > 6.5 mm	(17.6 - 71.1)	(81.8 - 91.9)	(11.3 - 34.8)	(92.7 - 96.9)	
Thickness bladder wall		30.0 (6.7 - 65.2)	72.2 (62.2 - 80.8)	10.0 (3.9 - 23.2)	90.9 (86.7 - 93.8)	1.1
Trabeculated bladder wall		33.3 (7.5 -70.0)	71.2 (61.0 - 80.1)	10.0 (4.0 - 22.8)	91.8 (87.4 - 94.7)	1.2
Post-void residual urine		50.0 (18.7 - 81.3)	45.4 (35.2 - 55.8)	8.6 (4.7 - 15.2)	89.8 (82.0 - 94.4)	0.7
Large post-void residual urine		30.0 (6.7 - 65.2)	61.8 (51.4 - 71.5)	7.5 (2.9 -17.7)	89.6 (84.7 -92.9)	2.9
Bladder capacity increased		22.2 (2.8 - 60.0)	78.7 (69.0 - 86.5)	9.0 (2.7 - 26.5)	91.3 (88.0 - 93.8)	1.0
Bladder capacity diminished		44.4 (13.7 - 78.8)	69.1 (58.8 -78.3)	12.1 (5.9 - 23.3)	92.8 (87.7 - 95.9)	1.8
Bladder capacity abnormal		66.7 (29.9 - 92.5)	47.9 (37.5 - 58.4)	10.9 (6.9 - 16.8)	93.7 (85.3 - 97.5)	1.8
Bladder diverticulum		10.0 (2.5 - 44.5)	84.5 (75.8 - 91.0)	6.2 (1.9 - 31.2)	90.1 (87.9 - 91.9)	0.6
RPD increased during voiding or detrusor contraction		42.6 (17.7 - 71.1)	92.5 (87.9 - 95.7)	28.6 (15.5 - 46.5)	95.9 (93.6 - 97.3)	9.2

95% CI = Confidence Interval 95%; PPV = positive predictive value; NPV = negative predictive value; DOR = diagnostic odds ratio; RPD = Renal pelvic diameter

### Renal damage

A total of 92 patients (86%) had information regarding renal scintigraphy (99mTc-DMSA). Twenty children, out of the 92, had renal damage (two bilateral), giving a total of 22 kidney units. The presence of renal damage was associated with high-grade reflux. Twelve units had high-grade reflux, 4 (33.3%) had renal damage, whereas, in 172 units with mild-moderate or absence of reflux, 18 (10.5%) had an abnormality on renal scintigraphy (P = 0.04). The presence of thinning of the renal parenchyma in DSUS predicted damage to renal scintigraphy. This finding presented a sensitivity of 40.9% (95% CI, 20.7 - 63.6), specificity of 94.4% (95% CI, 89.7 - 97.4), a PPV of 50% (95% CI, 30.8 - 69.2), an NPV of 92.2% (95% CI, 89.2 - 94.3), a DOR of 11.8, and an accuracy of 88% (95% CI, 82.5 - 92.3) for renal scarring.

## DISCUSSION

In this retrospective cohort study, we evaluated DSUS measurements as predictors for VUR and renal scarring in a cohort of children and adolescents with NB. Our findings showed that sonography kidney measurements predict with fair to good accuracy the presence of VUR and renal scarring, which are crucial to managing children with NB. Overall, the PPV was low due to the relatively low prevalence of VUR, but the NPV was high for all renal measurements.

Clinical studies have reported that secondary VUR is prevalent in children and adolescents with NB (8). For instance, Bortolini et al. (21) described results like ours (15.9%) and demonstrated 19% of VUR in patients with NB. Sidi et al. (22) showed a higher prevalence of 52% (46.7% high grade). We evaluated the performance of two continuous measurements, RPD and distal ureter diameter, in predicting VUR. As previously mentioned, the magnitude of both measurements could indicate the presence of VUR. However, we described relatively low accuracy in identifying all grades of VUR, which did not improve for high-grade reflux. The literature is limited to specific ultrasound findings' contribution to predicting VUR in children with NB (13). Our findings agree with the study by Naseri et al. (19), who described that hydronephrosis (RPD ≥ 5mm) has low accuracy (0.65) for general VUR and does not improve for high-grade VUR (0.66) in patients without NB with UTI (1-18 years). On the other hand, a study demonstrated hydronephrosis in 28.8% of patients (1-144 months) with a first episode of febrile UTI and 18.5% with high-grade VUR (DOR 18.8) (23). Swanton et al. (24) showed the distal diameter of the ureter as a measure to predict VUR. In our analysis, the distal ureteral diameter (>6.5 mm) had relatively low accuracy (DOR 4.5) for general-grade VUR and slightly better accuracy (DOR 7.5) for high-grade VUR. This finding agrees with a recent study that shows the presence of hydroureteronephrosis evidenced low accuracy (0.67) for general VUR but became moderate (0.82) for high-grade VUR (19). Lee et al. (23) reported that the presence of a hydroureter ≥ 7 mm in children without NB with a first UTI had a DOR of 20.4 for high-grade VUR. Recent studies suggest that measurement of the distal ureteral diameter is objective and reliable and is more predictive of the clinical outcome, regardless of the grade of VUR (24, 25). Our findings showed a considerable improvement in overall performance when we combined the two measurements (RPD and distal ureter diameter), with a sensitivity of 92.9% and an NPV of 94.8% for high-grade VUR.

Regarding the categorical measurements, we emphasize that the overall performance was low for all measures, except for the increase in RPD in the dynamic phase of the test. One of the peculiarities of DSUS is the assessment of RPD during urination or detrusor contractions as an indirect sign of VUR (14). In our analysis, this finding demonstrated a good accuracy (89%) in predicting VUR.

DSUS has been used in our clinic since developing the technique in 2003 (14), including for diagnosing patients with non-neurogenic dysfunction (26). Filgueiras et al. (14) demonstrated that DSUS is a sensitive method and correlates well with urodynamic findings. In this sense, Bortolini et al. (21) showed excellent accuracy (90% accuracy, kappa coefficient of 0.8, p < 0.001) of DSUS in identifying detrusor overactivity in patients with NB due to myelomeningocele when compared to urodynamic testing. The DSUS, a noninvasive test, has guided us in the follow-up of children with NB, as it can anticipate clinical worsening and help us in decision-making.

One of the main goals in monitoring children with NB is to identify early changes in the upper urinary tract and thus prevent long-term kidney damage (2, 7). Renal scintigraphy (99mTc-DMSA) is the gold standard test for detecting renal scarring, present in 25% of children with spina bifida with some degree of VUR (27). Scar detection was observed in adults with spinal dysraphism in 10% by ultrasonography and 46% by renal scintigraphy (99mTc-DMSA). Renal injury has been associated with high-grade VUR (28). Finkelstein et al. (12) demonstrated low accuracy in detecting renal scars by ultrasonography. Our findings showed the presence of renal scarring in 19.2% of patients submitted to renal scintigraphy (99mTc-DMSA). DSUS showed that renal parenchymal thinning predicts renal scarring on renal scintigraphy with moderate accuracy (88%). In a previous study in our clinic, renal scarring was detected in 31.7% of patients, with bladder wall thickness in DSUS being a marginal risk factor of renal scarring (29).

Our study has limitations. First, it is a retrospective study with inherent issues concerning this design, such as missing data. In this regard, we had to exclude some patients from the analysis due to the incomplete registry of the index tests. In addition, we tried to minimize the DSUS and VCUG findings of interpretation variability with a highly trained team using the same methodology. Also, we tried to mitigate the risk of verification bias by selecting the indexes and reference tests at the closest intervals with blinded radiologists. Thus, even though it is a retrospective study, when we present the results of a non-invasive test, such as the DSUS, with the possibility of predicting VUR in children with NB, we believe that it can be of great value in managing these patients. Thus, this finding could help minimize harm to these children and adolescents with such a severe and complex condition, including risks of urinary tract infection, exposure to ionizing radiation, discomfort and anxiety during an invasive test such as the VCUG.

## CONCLUSIONS

Dynamic and static ultrasound predict vesicoureteral reflux and renal scarring in children with neurogenic bladder with fair to good accuracy, and all ultrasound measurements exhibited a high negative predictive value, meaning that the absence of these findings indicates the absence of vesicoureteral reflux clinically significant. The increase in renal pelvic diameter during urination or detrusor contractions proved to be the most accurate parameter to indicate the presence of vesicoureteral reflux in this study. The thickness of the renal parenchyma showed good accuracy for renal scarring. Thus, our findings suggest that dynamic and static ultrasound and voiding cystourethrography should be considered complementary in the initial approach for children and adolescents with neurogenic bladder.

## ABBREVIATIONS

NB: Neurogenic bladder
VUR: Vesicoureteral reflux
VCUG: Voiding cystourethrography.
DSUS: Dynamic and static ultrasound
RPD: Renal pelvic diameter
RPT: Renal parenchyma thickness
ROC: Receiver operating characteristic curve
AUC: Area under the ROC curve
IQ: Interquartile range
LR: Likelihood ratio
DOR: Diagnostic odds ratio
NPV: Negative predictive value
PPV: Positive predictive value
US: Ultrasound
99mTc-DMSA: 99-Technetium dimercaptosuccinic acid

## APPENDIX

### Supplement 1 - STARD (STAndards for the Reporting of Diagnostic accuracy studies) Checklist (15).

**Table:**

Section & Topic	No	Item	Reported on page
**TITLE OR ABSTRACT**			
	**1**	Identification as a study of diagnostic accuracy using at least one measure of accuracy	1
**ABSTRACT**			
	**2**	Structured summary of study design, methods, results, and conclusions (for specific guidance, see STARD for Abstracts)	1
**INTRODUCTION**			
	**3**	Scientific and clinical background, including the intended use and clinical role of the index test	2
	**4**	Study objectives and hypotheses	2
**METHODS**			
*Study design*	**5**	Whether data collection was planned before the index test and reference standard were performed (prospective study) or after (retrospective study)	3
*Participants*	**6**	Eligibility criteria	3
	**7**	On what basis potentially eligible participants were identified (such as symptoms, results from previous tests, inclusion in registry)	3
	**8**	Where and when potentially eligible participants were identified (setting, location and dates)	3
	**9**	Whether participants formed a consecutive, random or convenience series	3
*Test methods*	**10a**	Index test, in sufficient detail to allow replication	3-4
	**10b**	Reference standard, in sufficient detail to allow replication	4-5
	**11**	Rationale for choosing the reference standard (if alternatives exist)	4-5
	**12a**	Definition of and rationale for test positivity cut-offs or result categories of the index test, distinguishing pre-specified from exploratory	4-5
	**12b**	Definition of and rationale for test positivity cut-offs or result categories of the reference standard, distinguishing pre-specified from exploratory	4-5
	**13a**	Whether clinical information and reference standard results were available to the performers/readers of the index test	4-5
	**13b**	Whether clinical information and index test results were available to the assessors of the reference standard	4-5
*Analysis*	**14**	Methods for estimating or comparing measures of diagnostic accuracy	5
	**15**	How indeterminate index test or reference standard results were handled	5
	**16**	How missing data on the index test and reference standard were handled	5
	**17**	Any analyses of variability in diagnostic accuracy, distinguishing prespecified from exploratory	5
	**18**	Intended sample size and how it was determined	5
**RESULTS**			
*Participants*	**19**	Flow of participants, using a diagram	N/A
	**20**	Baseline demographic and clinical characteristics of participants	5-6 (Table 1)
	**21a**	Distribution of severity of disease in those with the target condition	6 -7
	**21b**	Distribution of alternative diagnoses in those without the target condition	6-7
	**22**	Time interval and any clinical interventions between index test and reference standard	6-7
*Test results*	**23**	Cross tabulation of the index test results (or their distribution) by the results of the reference standard	6-7
	**24**	Estimates of diagnostic accuracy and their precision (such as 95% confidence intervals)	6-7
	**25**	Any adverse events from performing the index test or the reference standard	6-7
**DISCUSSION**			
	**26**	Study limitations, including sources of potential bias, statistical uncertainty, and generalisability	7-10
	**27**	Implications for practice, including the intended use and clinical role of the index test	7-10
**OTHER INFORMATION**			
	**28**	Registration number and name of registry	N/A
	**29**	Where the full study protocol can be accessed	Supplementary information
	**30**	Sources of funding and other support; role of funders	FAPEMIG

N/A-not applicable

#### STARD 2015

##### AIM

STARD stands for "Standards for Reporting Diagnostic accuracy studies". This list of items was developed to contribute to the completeness and transparency of reporting of diagnostic accuracy studies. Authors can use the list to write informative study reports. Editors and peer-reviewers can use it to evaluate whether the information has been included in manuscripts submitted for publication.

##### EXPLANATION

A **diagnostic accuracy study** evaluates the ability of one or more medical tests to correctly classify study participants as having a **target condition.** This can be a disease, a disease stage, response or benefit from therapy, or an event or condition in the future. A medical test can be an imaging procedure, a laboratory test, elements from history and physical examination, a combination of these, or any other method for collecting information about the current health status of a patient.

The test whose accuracy is evaluated is called **index test**. A study can evaluate the accuracy of one or more index tests. Evaluating the ability of a medical test to correctly classify patients is typically done by comparing the distribution of the index test results with those of the **reference standard**. The reference standard is the best available method for establishing the presence or absence of the target condition. An accuracy study can rely on one or more reference standards.

If test results are categorized as either positive or negative, the cross tabulation of the index test results against those of the reference standard can be used to estimate the **sensitivity** of the index test (the proportion of participants *with* the target condition who have a positive index test), and its **specificity** (the proportion *without* the target condition who have a negative index test). From this cross tabulation (sometimes referred to as the contingency or "2×2" table), several other accuracy statistics can be estimated, such as the positive and negative **predictive values** of the test. Confidence intervals around estimates of accuracy can then be calculated to quantify the statistical **precision** of the measurements.

If the index test results can take more than two values, categorization of test results as positive or negative requires a **test positivity cut-off**. When multiple such cut-offs can be defined, authors can report a receiver operating characteristic (ROC) curve which graphically represents the combination of sensitivity and specificity for each possible test positivity cut-off. The **area under the ROC curve** informs in a single numerical value about the overall diagnostic accuracy of the index test.

The **intended use** of a medical test can be diagnosis, screening, staging, monitoring, surveillance, prediction or prognosis. The **clinical role** of a test explains its position relative to existing tests in the clinical pathway. A replacement test, for example, replaces an existing test. A triage test is used before an existing test; an add-on test is used after an existing test.

Besides diagnostic accuracy, several other outcomes and statistics may be relevant in the evaluation of medical tests. Medical tests can also be used to classify patients for purposes other than diagnosis, such as staging or prognosis. The STARD list was not explicitly developed for these other outcomes, statistics, and study types, although most STARD items would still apply.

##### DEVELOPMENT

This STARD list was released in 2015. The 30 items were identified by an international expert group of methodologists, researchers, and editors. The guiding principle in the development of STARD was to select items that, when reported, would help readers to judge the potential for bias in the study, to appraise the applicability of the study findings and the validity of conclusions and recommendations. The list represents an update of the first version, which was published in 2003.

More information can be found on http://www.equator-network.org/reporting-guidelines/stard.

### Supplement 2 - Propaedeutic Protocol for Neurogenic Bladder in Pediatrics (1-3).

**Propaedeutics to confirm the diagnosis and establish the status of the neurogenic bladder.**
Apply a structured questionnaire, including questions about bladder emptying, urination, urinary leakage, use of catheterization and bladder drainage (daily filling of the bladder), and bowel function. Perform a complete physical examination with an evaluation of the external genitalia, lumbosacral region, and neurological reflexes. Laboratory tests: blood gas analysis, serum creatinine, urea, electrolytes, urinalysis, and urine culture Dynamic and static ultrasound (DSUS). Urodynamic study and voiding cystourethrography (VCUG) will be performed six weeks after surgery to correct spinal dysraphism due to spinal shock phase evaluation. If the correction of dysraphism is intrauterine, it will not be necessary to wait six weeks. Renal scintigraphy (99mTc-DMSA).
**Propaedeutics to follow-up**
Clinical examination, including clean bladder catheterization technique evaluation by nursing staff every six months. Laboratory tests: blood gas analysis, serum creatinine, urea, electrolytes, urinalysis, and urine culture every six months. Six-monthly DSUS through age two years and after that, annually or as clinically necessary and as soon as possible in the case of patients who have abandoned treatment and follow-up. Urodynamic study annually and as soon as possible in the case of patients who have abandoned treatment and follow-up. VCUG depends on the clinical evolution and will be repeated annually in the patient with high-grade VUR before urological surgery and as soon as possible in the case of patients who have abandoned treatment and follow-up. Renal scintigraphy (99mTc-DMSA) depends on the clinical and ultrasonographic evolution. Patients with worsening the neurological, orthopedic, or urological status, a neurosurgical evaluation should be performed (risk of symptomatic chord, syringomyelia, increased intracranial pressure caused by valve system dysfunction).
**Follow-up propaedeutics for bladder augmentation surgery using small bowel, colon, or gastric segments**

## References

[1] Bauer SB, Austin PF, Rawashdeh YF, Jong TP, de Franco I, Siggard C (2012). International Children's Continence Society's recommendations for initial diagnostic evaluation and follow-up in congenital neuropathic bladder and bowel dysfunction in children. Neurourol Urodyn.

[2] Stein R, Bogaert G, Dogan HS, Hoen L, Kocvara R, Nijman RJM (2020). EAU/ESPU guidelines on the management of neurogenic bladder in children and adolescent part I diagnostics and conservative treatment. Neurourol Urodyn.

[3] Sager C, Barroso U Jr, Bastos JM Netto, Retamal G, Ormaechea E. (2022). Management of neurogenic bladder dysfunction in children update and recommendations on medical treatment. Int Braz J Urol.

[4] Torre M, Buffa P, Jasonni V, Cama A (2008). Long-term urologic outcome in patients with caudal regression syndrome, compared with meningomyelocele and spinal cord lipoma. J Pediatr Surg.

[5] Bauer S, Belman AB, King LR, Stephen AK (2002). Neuropathology of the lower urinary tract. Clinical pediatric urology.

[6] de Azevedo RV, Oliveira EA, Vasconcelos MM, de Castro BA, Pereira FR, Duarte NF (2014). Impact of an interdisciplinary approach in children and adolescents with lower urinary tract dysfunction (LUTD). J Bras Nefrol.

[7] Sturm RM, Cheng EY (2016). The Management of the Pediatric Neurogenic Bladder. Curr Bladder Dysfunct Rep.

[8] Wu CQ, Franco I (2017). Management of vesicoureteral reflux in neurogenic bladder. Investig Clin Urol.

[9] El-Desoky SM, Banakhar M, Khashoggi K, Zaher ZF, Albanna AS, Kari JA (2022). Outcome of Urinary Bladder Dysfunction in Children. Indian J Pediatr.

[10] Silva JM, Diniz JS, Lima EM, Pinheiro SV, Marino VP, Cardoso LS (2009). Independent risk factors for renal damage in a series of primary vesicoureteral reflux: a multivariate analysis. Nephrology (Carlton).

[11] Khoury AE, Bagli DJ, Wein AJ, Kavoussi LR, Partin AW, Peters CA (2016). Vesicoureteral Reflux. Campbell-Walsh Urology.

[12] Finkelstein JB, Rague JT, Chow J, Venna A, Logvinenko T, Nelson CP (2020). Accuracy of Ultrasound in Identifying Renal Scarring as Compared to DMSA Scan. Urology.

[13] Logvinenko T, Chow JS, Nelson CP (2015). Predictive value of specific ultrasound findings when used as a screening test for abnormalities on VCUG. J Pediatr Urol.

[14] Filgueiras MF, Lima EM, Sanchez TM, Goulart EM, Menezes AC, Pires CR (2003). Bladder dysfunction: diagnosis with dynamic US. Radiology.

[15] Cohen JF, Korevaar DA, Altman DG, Bruns DE, Gatsonis CA, Hooft L (2016). STARD 2015 guidelines for reporting diagnostic accuracy studies: explanation and elaboration. BMJ Open.

[16] Dinkel E, Ertel M, Dittrich M, Peters H, Berres M (1985). Kidney size in childhood. Sonographical growth charts for kidney length and volume. Pediatr Radiol.

[17] Barry BP, Hall N, Cornford E, Broderick NJ, Somers JM, Rose DH (1998). Improved ultrasound detection of renal scarring in children following urinary tract infection. Clin Radiol.

[18] Lebowitz RL, Olbing H, Parkkulainen KV, Smellie JM, Tamminen-Mö;bius TE (1985). International system of radiographic grading of vesicoureteric reflux. International Reflux Study in Children. Pediatr Radiol.

[19] Naseri M, Karimi M, Bakhtiari E, Tafazoli N, Alamdaran SA, Tafazoli N (2021). Diagnostic Values of Kidney Ultrasonography for Vesicoureteral Reflux (VUR) and High Grade VUR. Iran J Kidney Dis.

[20] Weinstein S, Obuchowski NA, Lieber ML (2005). Clinical evaluation of diagnostic tests. AJR Am J Roentgenol.

[21] Bortolini T, Lucena IRS, da Silva Batezini NS, Rosito TE, Araújo T, Carneiro BB (2019). Can dynamic ultrasonography replace urodynamics in the follow-up of patients with myelomeningocele? A prospective concurrent study. Neurourol Urodyn.

[22] Sidi AA, Peng W, Gonzalez R (1986). Vesicoureteral reflux in children with myelodysplasia: natural history and results of treatment. J Urol.

[23] Lee JH, Kim MK, Park SE (2012). Is a routine voiding cystourethrogram necessary in children after the first febrile urinary tract infection?. Acta Paediatr.

[24] Swanton AR, Arlen AM, Alexander SE, Kieran K, Storm DW, Cooper CS (2017). Inter-rater reliability of distal ureteral diameter ratio compared to grade of VUR. J Pediatr Urol.

[25] Payza AD, Hocgö;r M, Serdaroğlu E, Sencan A (2019). Can distal ureteral diameter measurement predict primary vesicoureteral reflux clinical outcome and success of endoscopic injection?. J Pediatr Urol.

[26] Pinto FNCS, de Bessa J, Bastos JM, Dias GCM, Vasconcelos MMA, Lima EM (2023). Validation of the Vancouver Symptom Score Questionnaire for bladder and bowel dysfunction for Brazilian children and adolescents. Int Braz J Urol.

[27] Shiroyanagi Y, Suzuki M, Matsuno D, Yamazaki Y (2009). The significance of 99mtechnetium dimercapto-succinic acid renal scan in children with spina bifida during long-term followup. J Urol.

[28] Veenboer PW, Hobbelink MG, Ruud Bosch JL, Dik P, van Asbeck FW, Beek FJ (2015). Diagnostic accuracy of Tc-99m DMSA scintigraphy and renal ultrasonography for detecting renal scarring and relative function in patients with spinal dysraphism. Neurourol Urodyn.

[29] Leonardo CR, Filgueiras MF, Vasconcelos MM, Vasconcelos R, Marino VP, Pires C (2007). Risk factors for renal scarring in children and adolescents with lower urinary tract dysfunction. Pediatr Nephrol.

